# Supplementary material for: Does Pancreatic Fistula Affect Long-Term Survival after Resection for Pancreatic Cancer? A Systematic Review and Meta-Analysis
Source: Cancers (Basel). 2021 Nov 19;13(22):5803. doi: 10.3390/cancers13225803 (PMC8616191; doi:10.3390/cancers13225803)
Supplement: Supplementary file 1 [file cancers-13-05803-s001.zip › cancers-1416999-supplementary.pdf]

TABLES

Table S1. Search strategy

Sites	Search terms
MEDLINE, Embase, Web of Science and Cochrane Library	(pancreatic ductal adenocarcinoma **OR** pancreatic adenocarcinoma **OR** pancreatic cancer **OR** pancreatic carcinoma) **AND** (pancreatic fistula **OR** pancreatic fistula* **OR** pancreatic leak **OR** POPF) **AND** (survival **OR** long-term survival **OR** long term survival **OR** disease-free survival **OR** recurrence-free survival)
Scopus	(**TITLE-ABS-KEY** ("pancreatic ductal adenocarcinoma" **OR** "pancreatic adenocarcinoma" **OR** "pancreatic cancer" **OR** "pancreatic carcinoma") **AND TITLE-ABS-KEY** ("pancreatic fistula" **OR** "pancreatic fistula*" **OR** leak **OR** POPF) **AND TITLE-ABS-KEY** (survival **OR** "long-term survival" **OR** "long term survival" **OR** "disease-free survival" **OR** "recurrence-free survival")

Table S2. General characteristics of the studies and the patients considered in the systematic review

Author	NAT	Study type	Country	Study duration (Follow-up)	Age (range)	Male sex n (%)
Ausania 2010 [14]	NO	RCS	UK	2002-2007 (30.8 m)	NA	27 (57.4)
Nagai 2011 [15]	NO	RCS	Japan	2001-2009	63.9 (35-83)	108 (58.7)
Ren 2011 [16]	NA	RCS	China	1994-2007	NA	95 (59.4)
Assifi 2013 [17]	NO	RCS	USA	2001-2009 (36 m)	66 (35-91)	114 (51.6)
Murakami 2015 [18]	NO	RCS	Japan	1996-2013 (48 m)	69 (31-88)	116 (55)
Kawai 2016 [19]	YES+NO	mRCS	Japan	2001-2012 (25.3 m)	66 (27-91)	767 (54.9)
Lin 2016 [20]	NA	RCS	China	1995-2003	NA	162 (54)
Watanabe 2017 [21]	NO	RCS	Japan	1996 - 2004 (to December 2015)	69 (43-84)	74 (61)
Dundar 2018 [22]	NO	RCS	Turkey	2007-2015	61 (39-84)	44 (68.75)
Christos 2019 [23]	NA	RCS	Greece	2004-2015	71 (31-89)	124 (54.9)

Author	NAT (n)	Study type	Country	Study duration (Follow-up)	Age	Sex male n (%)
Hank 2019 [24]	YES (346)	RCS	USA	2007-2017 (16 m)	66 (59-72)	163 (47.1)
	NO				70 (62-77)	201 (49.4)
Neeman 2020 [25]	YES+NO	RCS	Israel	2008-2016 (22 m)	66 (41-85)	88 (59)
Uchida 2020 [26]	YES (52)	RCS	Japan	2012-2019	67.5 (49-85)	33 (63)
	NO (148)				71 (48-89)	92 (62)
Bonaroti 2021 * [27]	YES+NO	RCS	USA	2009-2016	NA	NA
Dhayat 2021 * [28]	NO	RCS	Germany	2007-2016	NA	NA
Leon 2021 [29]	YES+NO	mRCS	EU	2008-2017 (24 m)	70 (37-88)	139 (49.1)

NAT neoadjuvant therapy; EU, Europe; mRCS, multicentric retrospective cohort study; NA, not available; RCS, retrospective cohort study; UK, United Kingdom; USA, United States of America * only data relating to pancreatic adenocarcinoma were extracted

Table S3. Disease-free survival: hazard ratios and 95% confidence intervals for patients with/without pancreatic fistula

		Univariate			Multivariate			
Author	NAT (n)	All POPF vs none HR (95% CI)	POPF B+C vs none HR (95% CI)	p value	All POPF vs none HR (95% CI)	POPF B+C vs none HR (95% CI)	p value	Adjusted factor in multivariate analysis
Ausania 2010 [14]	NO							
Nagai 2011 [15]	NO					1.19 (0.77-1.85)	0.427	IORT, ACT, T, N, G, R1
Ren 2011 [16]	NA							
Assifi 2013 [17]	NO	1.22 (0.76-1.96)		0.42	1.04 (0.56-1.91)		0.9	VI, Stage, ACT, RT, G
Murakami 2015 [18]	NO							
Kawai 2016 [19]	YES+NO				GRADE B<br>GRADE C	1.41 (0.94-2.13)	0.1	Blood transfusion, Stage, R1, VI, N, ACT
Lin 2016 [20]	NA							
Watanabe 2017 [21]	NO		1.86 (1.17-2.91)	0.01				
Dundar 2018 [22]	NA	1.63(1.14-2.32) *		0.007				
Cristhos 2019 [23]	NA			0.634	1.011 (0.7-1.46) §		0.916	T, Stage, NI, N, VI, R1, CA 19-9
Hank 2019 [24]	YES (346)							
	NO							
Neeman 2020 [25]	YES+NO	1.06 (0.67-1.65)	1.14 (0.67-197)	0.80<br>0.629	1.2 (0.73-1.95)		0.462	ACT, NI, N, G
Uchida 2020 [26]	YES (52)		2.82 (1.04-6.46)	0.023		5.86 (1.10-15.48)	0.002	Age, N
	NO (148)			0.766				
Bonaroti 2021 [27]	YES+NO		1.45 (0.85-2.48)	0.175				
Dhayat 2021 [28]	NO		3.8 (1.99-7.25)	<0.001		1.88 (1.19-2.95)	0.006	NYHA, CA 19-9, T, G, Stage, N, POC
Leon 2021 [29]	YES+NO		1.34 (9.92-1.96)	0.121		1.44 (0.92-2.25)	0.107	WL, NAT, CA 19-9, T, N, R1, VI

ACT, adjuvant chemotherapy; All POPF, postoperative pancreatic fistula grades A+B+C; CA 19-9, preoperative CA 19-9 level; G, tumor grade; HR, hazard ratio; IORT, intraoperative radiotherapy; NA, not available; n, number of patients; N, nodes (TNM); NAT, neoadjuvant therapy; NI, neural invasion; NYHA, New York Heart Association (classification); POC, postoperative complication; POPF, postoperative pancreatic fistula; RT, radiotherapy; R1, residual tumor; SES, socioeconomic status; SM, smoking; T, tumor (TNM); VI, vascular invasion; WL, preoperative weight loss; 95% CI, 95% confidence interval; § 95% CI, 95% confidence interval estimated from p; * HR and 95% CI extracted from median survival.

Table S4. Overall survival: hazard ratios and 95% confidence intervals for patients with/without pancreatic fistula

		Univariate			Multivariate			
Study name	NAT (n)	All POPF vs none HR (95% CI)	POPF B/C vs none HR (95% CI)	p value	All POPF vs none HR (95% CI)	POPF B/C vs none HR (95% CI)	p value	Adjusted factor in multivariate analysis
Ausania 2010 [14]	NO	1.66(0.5-5.52) *		0.411				
Nagai 2011 [15]	NO		1.1 (0.5-2.39) *	0.83	POPF GRADE B+C			
Ren 2011 [16]	NA	1.35 (0.48-3.81) §		0.582				
Assifi 2013 [17]	NO	1.17 (0.72-1.91)		0.52	1.08 (0.57-2.04)		0.82	VI, Stage, ACT. RT, G
Murakami 2015 [18]	NO	1.11(0.62-1.98) *		0.743				
Kawai 2016 [19]	NA				POPF GRADE B<br>POPF GRADE C	0.76 (0.56-1.03)<br>1.59 (1.03-2.45)	0.079<br>0.035	Age, blood loss, Stage, VI, N, R1, ACT, POC
Lin 2016 [20]	NA	1.9 (1.38-2.64) *		<0.0001	POPF GRADE B<br>POPF GRADE C	2.23 (1.35-3.7)<br>2.92 (1.44-5.93)	0.0018<br>0.003	SES, Ca 19-9, N, POC WL, SM
Watanabe 2017 [21]	NO		1.66 (1.03-2.6)	0.04				
Dundar 2018 [22]	NA	1.47 (0.84-2.59) *		0.18				
Cristhos 2019 [23]				0.818	1.05 (0.7-1.56)		0.901	Age, T, G, NI, R1 …
Hank 2019 [24]	YES			0.002		2.8 (1.44-5.45)	0.002	T, N, G, R1, ACT,
	NO		1.04 (0.88-1.23)	0.66		NA	NA	CA 19-9
Neeman 2020 [25]	YES+NO	0.99 (0.61-1.59)	1.01 (0.57-1.81) *	0.94/0.96	0.99 (0.59-1.64)		0.96	ACT, R1, N, G, NI
Uchida 2020 [26]	YES (52)		5.36 (1.45-16.33)	0.005		7.1 (1.39-31.54)	0.02	POC, N
	NO			0.863				
Bonaroti 2021 [27]	YES+NO		1.17 (1.03-1.34) *	0.002	Grade B<br>Grade C	1.52 (1.09-2.12)<br>2.92 (1.43-5.95)	0.013<br>0.003	ASA, albumin, TNM, R1, VI,
Dhayat 2021 [28]	NO		2.76 (1.45-5.26)	0.002		1.28 (0.71-2.32)	0.408	NYHA, CA 19-9, T, G, Stage, N, POC
Leon 2021 [29]	YES+NO		1.31 (0.85-2.02)	0.226				ASA, Age, WL, CA 19-9 T, N, G, VI

ACT, adjuvant chemotherapy; All POPF, postoperative pancreatic fistula grades A+B+C; ASA, American Society of Anesthesiology score ; CA 19-9, preoperative CA 19-9 level; G, tumor grade; HR, hazard ratio; NA, not available; n, number of patients; N, nodes (TNM); NAT, neoadjuvant therapy; NI, neural invasion; NYHA, New York Heart Association (classification); POC, postoperative complication; POPF, postoperative pancreatic fistula; RT, radiotherapy; R1, residual tumor; SES, socioeconomic status; SM, smoking; T, tumor (TNM); VI, vascular invasion; WL, preoperative weight loss; 95% CI, 95% confidence interval; § 95% CI, 95% confidence interval estimated from p; * HR and 95% CI extracted from median survival.

Supplemental Table S5. Detailed scores on the Newcastle Ottawa Scale for each cohort study considered

Author	Selection				Comparability	Outcome		
	Representative-ness of intervention cohort	Selection of non-intervention cohort	Ascertainment of intervention	Demonstration that outcome of interest was not present at start of study	Comparability of cohorts on the basis of the design or analysis	Assessment of outcome	Length of follow-up	Adequacy of follow-up of cohorts
**Ausania 2010 [14]**	1	1	1	0	2	1	1	1
**Nagai 2011 [15]**	1	1	1	0	2	1	1	1
**Ren 2011 [16]**	1	1	1	0	1	1	1	1
**Assifi 2013 [17]**	1	1	1	0	2	1	1	1
**Murakami 2015 [18]**	1	1	1	0	1	1	1	1
**Kawai 2016 [19]**	1	1	1	0	1	1	1	1
**Lin 2016 [20]**	1	1	1	0	1	1	1	1
**Watanabe 2017 [21]**	1	1	1	0	2	1	1	1
**Dundar 2018 [22]**	1	1	1	0	2	1	1	1
**Cristhos 2019 [23]**	1	1	1	0	1	1	1	1
**Hank 2019 [24]**	1	1	1	0	2	1	1	1
**Neeman 2020 [25]**	1	1	1	0	1	1	1	1
**Uchida 2020 [26]**	1	1	1	0	2	1	1	1
**Bonaroti 2021 [27]**	1	1	1	0	1	1	1	1
**Dhayat 2021 [28]**	1	1	1	0	1	1	1	1
**Leon 2021 [29]**	1	1	1	0	2	1	1	1

FIGURES

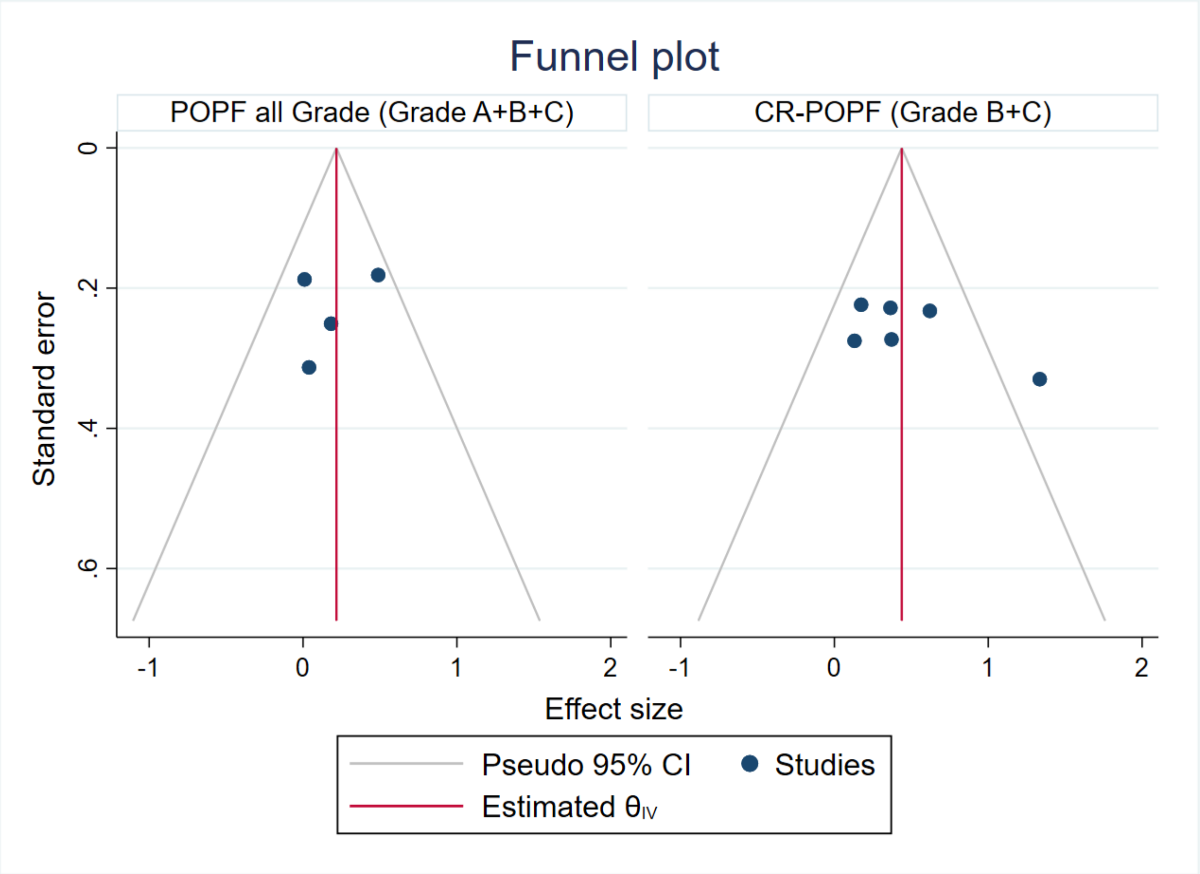

Figure S1. Funnel plot of meta-analysis reporting disease-free survival by grade of postoperative pancreatic fistula (POPF). CR-POPF, clinically-relevant POPF.

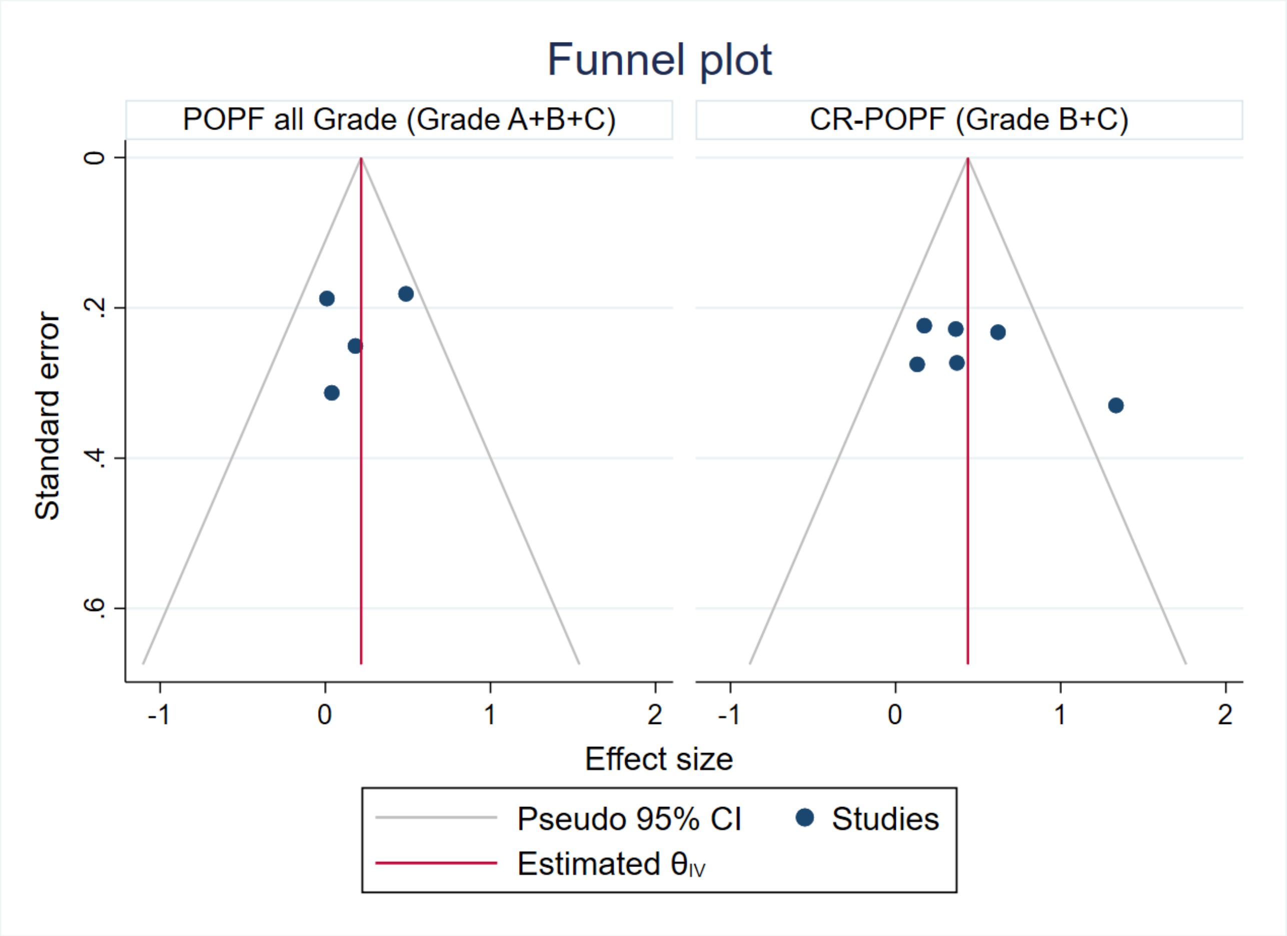

Figure S2. Funnel plot of meta-analysis reporting overall survival by grade of postoperative pancreatic fistula (POPF), and neoadjuvant (NAT) pretreatment. CR-POPF, clinically-relevant POPF.
